# Burden of Tuberculosis in Western Sudan During the Sudan Armed Conflict

**DOI:** 10.7759/cureus.76944

**Published:** 2025-01-05

**Authors:** Amal Khalil Y Mohammed, Eldisugi Hassan M Humida, Ahmed Mirghani O Ali, Hussain G Ahmed

**Affiliations:** 1 Medicine, Faculty of Medicine, University of Kordofan, El-Obeid, SDN; 2 Medicine, El-Obeid International Hospital, El-Obeid, SDN; 3 Cardiac Catheterization Laboratory, El-Obeid International Hospital, El-Obeid, SDN; 4 Medicine, El-Obeid Teaching Hospital, El-Obeid, SDN; 5 Histopathology and Cytology, Faculty of Medical Laboratory Sciences, University of Khartoum, Khartoum, SDN; 6 Pathology, Prof Medical Research Consultancy Center, El-Obeid, SDN

**Keywords:** infectious diseases, multidrug-resistant, mycobacterium tuberculosis, sudan, tuberculosis

## Abstract

Background: Tuberculosis remains the leading cause of death from infectious diseases worldwide. This study assesses the impact of tuberculosis in western Sudan during armed conflict in 2023-2024.

Methodology: This study was conducted by the respiratory department of El-Obeid Teaching Hospital in North Kordofan State, Sudan. The study encompassed 751 patients with tuberculosis. Data were collected in August 2024. A data collection sheet was developed to compile all pertinent information.

Results: Of the 751 patients, 659 (87.7%) were classified as new cases, 85 (11.3%) as recurrent cases, and seven (9.3%) as multidrug-resistant (MDR). Of the 659 new cases, 470 (86.2%) were male patients, and 206 (91.7%) were female. The majority of patients were aged 26-35 years with 166 (22.1%), followed by those aged 19-25 years at 160 (21.3%) and 36-45 years at 127 (16.9%), totaling 127 individuals.

Conclusion: The incidence rate of tuberculosis rose markedly during the armed conflict in Sudan from 54 per 100,000 to 63 per 100,000 people. During wartime, there was an increase in recurrent disease, non-compliance with treatment methods, and multidrug resistance. The demographics most affected include men, laborers, and individuals employed in gold mining. Extrapulmonary tuberculosis is increasingly detected in lymph nodes, pleura, and abdominal areas. Immediate enhancements to the healthcare system are necessary to prevent the widespread transmission of tuberculosis in Sudan.

## Introduction

Tuberculosis (TB) has been linked to poverty, starvation, overcrowding, and immunosuppression since the beginning of recorded history [1]. TB is the leading infectious disease killer of adults globally, with over 10 million new cases each year [2]. Diagnostic advances such as quick molecular testing (e.g., GeneXpert MTB/RIF, Cepheid Inc., Sunnyvale, California) and whole-genome sequencing in sputum and non-sputum material could change this [2]. Africa has the greatest human immunodeficiency virus (HIV) prevalence and TB/HIV co-infection (31.25%). TB has become more prevalent among immigrants and socially disadvantaged individuals in wealthy countries. Extrapulmonary presentation increases with non-European ancestry, HIV infection, and younger age [1].

*Mycobacterium tuberculosis* (MTB) kills 1.6 million people annually [3]. A neutrophil-driven type I interferon (IFN) signature is associated with active TB, although the molecular mechanisms of pathogenesis are not fully known [3]. Multidrug-resistant tuberculosis (MDR-TB) is a global health threat that is costly to patients and their families. Due to its prolonged treatment duration and expensive second-line therapy, MDR-TB is believed to cost more than drug-susceptible TB. However, the financial burden of MDR-TB is still not fully known. MDR-TB patients spent more on direct than indirect costs. The patients and families need social protection and financial support to minimize MDR-TB's devastating economic effects [4].

In 2018, the WHO recommended all-oral therapy for drug-resistant TB for the first time due to improved efficacy using new and repurposed medications. Shorter latent TB prevention regimens with rifampicin or rifapentine are equally successful as longer isoniazid-based regimens, and a vaccine candidate may prevent disease progression. New tools alone are insufficient. High-quality, people-centered TB care must improve. Renewing political commitment and improving care could potentially eliminate TB morbidity, death, and stigma [2].

A Sudanese meta-analysis examined pulmonary TB prevalence in 16 studies. The pooled prevalence was 30.72% (95% confidence interval (CI): 30.64, 30.81). Based on 2,737 participants, Khartoum State had the highest pooled prevalence of 41.86% (CI: 14.69, 69.02). Furthermore, TB significantly correlated with male gender and rural residency [5]. The present study assesses western Sudan's TB burden during the Sudan conflict (2023-2024).

## Materials and methods

This retrospective descriptive study was conducted in the respiratory department of El-Obeid Teaching Hospital located in North Kordofan State, Sudan. The investigation included 751 patients diagnosed with TB, along with comprehensive coverage files. Data collection took place in August 2024. This encompassed patients enrolled from August 5, 2023, to August 5, 2024. All patients exhibiting clinical symptoms indicative of TB and diagnosed with either pulmonary or extrapulmonary TB were included. Individuals exhibiting symptoms akin to TB who were not verified as TB patients were excluded.

A detailed data collection sheet was developed, and all pertinent information was thoroughly retrieved. Variables pertaining to each patient were extracted from the patient's records at the TB unit of El-Obeid Teaching Hospital. The retrieved variables encompassed the patient's demographic data, clinical symptoms, X-rays, and laboratory test results.

The demographic characteristics comprised age, sex (male, female), and occupations (employees, farmers, gold miners, healthcare workers, unemployed individuals, soldiers, students, and laborers). The clinical features included signs and symptoms comprising the presence of fever, cough, and significant loss of appetite. Accordingly, patients were categorized into new cases (first-time patients), recurrent cases (patients previously treated for TB who have been reinfected), and MDR cases (cases exhibiting resistance to available anti-TB medications).

Laboratory analyses

The laboratory analyses incorporated were the sputum Ziehl-Neelsen stain (ZN) assay for pulmonary TB and the GeneXpert, which is a cartridge-based nucleic acid amplification test (NAAT) designed for the swift diagnosis of TB and the rapid assessment of drug sensitivity. This is an automated diagnostic assay capable of detecting MTB DNA and resistance to rifampicin (RIF).

Samples tested

Tissue biopsy is used to diagnose extrapulmonary TB and certain cases of pulmonary TB. Fluids include pleural effusion, pericardial effusion, and cerebrospinal fluid ascites. Other variables were also assessed, including patient adherence to treatment protocol, causes of treatment delay, and causes of re-treatment.

Statistical analysis

We imported data sets using IBM SPSS Statistics for Windows, Version 25 (Released 2017; IBM Corp., Armonk, New York), and subsequently generated results. Cross-tabulations, frequencies, percentages, and means were calculated.

## Results

A total of 751 TB patients were admitted to our hospital, comprising 545 males (72.6%) and 206 females (27.4%). The age range of the patients was 1-90 years, with a mean age of 38 years. Among the 751 patients, 659 (87.7%) were identified as new cases (came for the first time), 85 (11.3%) were recurrent cases, and seven (9.3%) were classified as MDR (recurring cases). Among the 659 new cases, 470 out of 545 (86.2%) were male, while 189 out of 206 (91.7%) were female. The relative risk (RR) and 95% confidence interval (95%CI) for new TB cases in female patients are as follows: RR (95%CI) = 1.1494 (1.0154 to 1.3011), P = 0.0277.

Regarding patient adherence to the treatment protocol, only 13 out of 751 (1.7%) patients exhibited non-compliance due to the complexities of the armed conflict hindering their access to assistance. Regarding treatment outcomes, approximately 348 out of 751 (46.3%) completed the treatment (excluding recurrence assessment), 151 (20.1%) were on medication, 123 (16.3%) were cured (tested negative for TB with the elimination of signs and symptoms), 72 (9.5%) died, 51 (6.7%) defaulted (lost to follow-up), and six (0.8%) were transferred out (transferred to hospitals in another state), as illustrated in Table 1.

**Table 1:** The distribution of patients based on treatment issues MDR: multidrug-resitant

Variable	New Cases	Recurrent	MDR	Total
Patient adherence to the treatment protocol				
Not adherent	3	10	0	13
Adherent	656	75	7	738
Total	659	85	7	751
Treatment outcome				
Cured	106	13	4	123
Completed	319	29	0	348
Transferred out	4	1	1	6
Defaulted	45	6	0	51
Died	66	5	1	72
On medication	119	31	1	151
Total	659	85	7	751

Table 2 summarizes the distribution of patients by outcomes and adherence to treatment issues. Among the 13 patients who did not adhere to the treatment, four (30.7%) succumbed. Approximately 677 (90%) patients experienced a delay in treatment, with 266 (39.3%) reporting financial difficulties, 235 (34.7%) reporting security concerns, and 176 (26%) reporting a lack of health services as the causes for the treatment delay. Relapse is the primary cause of retreatment, accounting for 67 (88.2%) out of 76 cases (retreated cases), followed by eight (10.5%) defaulted cases and one (1.3%) failure. Approximately 74 patients were previously misdiagnosed, having presented before the conflict without confirmation of TB (included with the new cases).

**Table 2:** The distribution of patients based on their outcomes and adherence to treatment issues

Variable	Cured	Completed	Transferred Out	Defaulted	Died	On Medication	Total
Patient adherence to the treatment protocol							
Not adherent	1	5	1	2	4	0	13
Adherent	122	343	5	49	68	151	738
Total	123	348	6	51	72	151	751
Causes of delayed treatment							
No service	34	103	2	16	14	7	176
Security issues	29	95	2	19	21	69	235
Financial issues	46	108	0	11	30	71	266
Missed diagnosed	14	42	2	5	7	4	74
Total	123	348	6	51	72	151	751
Causes of re-treatment							
New cases	114	323	5	46	67	120	675
Failure	0	1	0	0	0	0	1
Defaulter	1	4	1	0	1	1	8
Relapse	8	20	0	5	4	30	67
Total	123	348	6	51	72	151	751

## Discussion

The interplay of poverty, chronic disease, and limited access to medical education significantly exacerbates the overall burden of TB in Sudan, particularly in the context of the ongoing armed conflict [6]. The health system has been severely impacted, especially in western Sudan, leading to a significant rise in poverty levels. The inability to access health services due to security concerns has resulted in the loss of many patients' lives [7]. The prevalence of TB is significantly greater in vulnerable populations compared to the general population, indicating a necessity for enhanced integration of these groups. This includes focused initiatives for their identification, targeted screening, and prevention strategies, along with support for treatment [8].

This study examines the burden of TB within a specific city and a hospital that provides TB diagnosis and treatment services. The results of the current study indicated that the occurrence of new cases was 87.7% of all TB cases, with a prevalence of pulmonary TB at 68.9%. A study in Sudan found that 73.4% of cases were pulmonary TB, while 26.6% of all TB patients had extrapulmonary TB [9]. Subsequent studies indicated an increased ratio of pulmonary TB among overall TB cases, potentially attributable to rising poverty and the deterioration of the health system due to conflict.

According to estimates, TB caused 1% of all inpatient deaths in Sudan in 2017. A meta-analysis conducted in Sudan found a pooled prevalence of 30.72% (CI: 30.64, 30.81). Additionally, Khartoum State exhibited the highest pooled prevalence at 41.86% (CI: 14.69, 69.02), derived from a total sample size of 2,737 participants [5]. The study examined the effects of the Sudan War on the well-being of children with pulmonary TB and the difficulties encountered by healthcare providers since the onset of the conflict. Before the conflict began, the pulmonary TB cure rate stood at 76.5%, with treatment success rates reaching 88.2% and a 6.1% mortality rate among children, many of whom were HIV positive [5,10].

In this study, the majority of extrapulmonary cases manifested as lymphadenopathy (11.9%), pleural effusion (8.7%), and abdominal involvement (4.3%). Previous reports have documented such findings. The most frequently observed locations are lymph nodes (19%), pleura (7%), gastrointestinal tract (4%), bone (6%), CNS (3%), and the genitourinary system (1%). The location of the disease influences the manifestations, complicating the diagnostic process as extrapulmonary TB can resemble various other conditions [11].

The results of the current investigation indicated that the majority of patients were male, comprising 72.6% of the sample. Occasionally, reports highlight the differences in incidence rates for TB between genders. Nonetheless, the extent and reliability of the variations across age groups, among diverse populations, and throughout prolonged durations remain ambiguous. It was previously reported that the incidence rates for TB exhibited gender differences, showing elevated rates in boys under one year old, no notable differences in boys aged one to nine, and increased rates in boys and men over the age of 15. The age bracket of 10-14 years accounted for the sole surplus in the female population. The observed age-related gender differences in TB incidence rates across various countries highlight the necessity of considering sex as a biological variable in the evaluation of TB risk factors [12].

The majority of patients in this series received their diagnoses through the use of both sputum for ZN and X-ray methods. The diagnosis of TB disease is fundamentally dependent on the identification or isolation of *M. tuberculosis* bacteria from a clinical sample. Smear microscopy for acid-fast bacilli (AFB), mycobacterial culture, or NAAT tests can be utilized to achieve this. The suitable sample will be determined by the suspected site of the disease. The quality of the sample can significantly influence the likelihood of obtaining a positive result; thus, it is essential to provide clear instructions to the patient on how to produce a sputum sample. Young children frequently struggle to produce sputum, making gastric aspirate a common necessity in these cases.

The most notable progress in TB diagnosis over the past 10 years has been the introduction of the GeneXpert MTB/RIF test. In 2013, the WHO released updated policy guidance regarding the use of Xpert MTB/RIF, further endorsing its application for extrapulmonary and pediatric samples. This policy change clarified that Xpert MTB/RIF should be used for samples from children and outside the lungs, such as gastric aspirate, lymph aspirate, pleural fluid, and cerebrospinal fluid. The available evidence was inadequate to assess sensitivity using urine, pericardial fluid, and ascitic fluid; however, specificity tends to be high for these sample types [13].

The results of this study indicated that approximately 11% of the patients experienced recurrent disease, a figure that is notably higher than previously reported values. The recurrence incidence was 8.3%, with 85.9% of these patients experiencing it within the one- to five-year time frame following successful treatment. It was previously reported that the recurrence rate showed no significant correlation with gender, age group, or diabetes status. However, it was notably elevated in individuals whose sputum smear grading prior to treatment was 2 + or greater, those with a positive sputum smear at the conclusion of the second month of treatment, individuals who had completed their treatment, and smokers (P < 0.05) [14].

Approximately 1% exhibited MDR. A study indicated that among the 10 million new cases of TB worldwide in 2017, approximately 558,000 were RIF, with 82% classified as MDR-TB. There were 5,102 documented cases in England, of which 55 (1.8%) were identified as MDR-TB. Cases of MDR-TB exhibit poorer outcomes and represent a significant challenge for public health. The management of MDR-TB continues to pose significant challenges, primarily due to the reliance on extended second-line drug therapies, which tend to be less effective and more toxic compared to first-line options. Two novel pharmacological interventions have received approval: bedaquiline and delamanide. Furthermore, a condensed treatment regimen lasting 9-12 months may be an alternative to the standard 20-24-month regimen [15].

Approximately 1.7% of the patients failed to comply with the treatment protocol. Following prescribed treatment is an essential aspect of clinical trials in TB treatment. Recent evidence indicates that adherence has a significant impact on therapy outcomes, warranting a greater focus on its quantification and implementation measures. The WHO organized a technical consultation that concentrated on innovations in clinical trial design for the creation of new TB treatments [16].

The WHO target regimen profiles (TRPs) indicate, according to expert opinion, that each element of the regimen should allow for a mutation rate not exceeding 1/10^7^ mutations per bacterium per generation. If the regimen is followed and there is no preexisting drug resistance, new resistance to one or more drugs in the regimen should develop in less than 2% of treatment courses. The WHO-recommended multiply MDR regimen, which uses five effective drugs, establishes this minimal target at an acquired resistance rate of 0%-2% [17].

There is a scarcity of epidemiological data from Sudan, notably from western Sudan, specifically North Kordofan State, where this study was conducted. According to the World Bank's compilation of development indicators derived from publicly available sources, the incidence of TB (per 100,000 people) in Sudan was 54 in 2022 [18]. Our recent study shows an incidence of 63 per 100,000 people, bearing in mind that the North Kordofan State of Sudan does not have the greatest prevalence of TB.

The results of this study indicate that men, laborers, and individuals involved in gold-mining activities exhibit higher infection rates. Laborers and individuals with lower socioeconomic status were reported to be more susceptible to TB infection [19].

Limitations

The current study provides important data on TB in Sudan during the ongoing conflict; however, it has limitations, including the lack of epidemiological data prior to the war and its retrospective design.

## Conclusions

The incidence rate of TB significantly increased during the armed conflict in Sudan from 54 per 100,000 to 63 per 100,000 people. The recurrence of disease, non-compliance with treatment protocols, and the rise of MDR strains escalated during the wartime period. The populations most impacted include males, laborers, and those engaged in gold-mining activities. The prevalence of extrapulmonary TB is on the rise, showing an increasing incidence in lymph nodes, pleura, and abdominal locations. Immediate actions are necessary within the health system to halt the devastating spread of TB in Sudan.
